# Bronchoconstriction Induces TGF-β Release and Airway Remodelling in Guinea Pig Lung Slices

**DOI:** 10.1371/journal.pone.0065580

**Published:** 2013-06-26

**Authors:** Tjitske A. Oenema, Harm Maarsingh, Marieke Smit, Geny M. M. Groothuis, Herman Meurs, Reinoud Gosens

**Affiliations:** 1 Department of Molecular Pharmacology, Groningen Research Institute for Asthma and COPD, University of Groningen, Groningen, The Netherlands; 2 Groningen Research Institute for Asthma and COPD (GRIAC), University of Groningen, Groningen, The Netherlands; 3 Department of Pharmacokinetics, Toxicology and Targeting, Groningen Research Institute of Pharmacy, University of Groningen, Groningen, The Netherlands; Helmholtz Zentrum München/Ludwig-Maximilians-University Munich, Germany

## Abstract

Airway remodelling, including smooth muscle remodelling, is a primary cause of airflow limitation in asthma. Recent evidence links bronchoconstriction to airway remodelling in asthma. The mechanisms involved are poorly understood. A possible player is the multifunctional cytokine TGF-β, which plays an important role in airway remodelling. Guinea pig lung slices were used as an *in vitro* model to investigate mechanisms involved in bronchoconstriction-induced airway remodelling. To address this aim, mechanical effects of bronchoconstricting stimuli on contractile protein expression and TGF-β release were investigated. Lung slices were viable for at least 48 h. Both methacholine and TGF-β_1_ augmented the expression of contractile proteins (sm-α-actin, sm-myosin, calponin) after 48 h. Confocal fluorescence microscopy showed that increased sm-myosin expression was enhanced in the peripheral airways and the central airways. Mechanistic studies demonstrated that methacholine-induced bronchoconstriction mediated the release of biologically active TGF-β, which caused the increased contractile protein expression, as inhibition of actin polymerization (latrunculin A) or TGF-β receptor kinase (SB431542) prevented the methacholine effects, whereas other bronchoconstricting agents (histamine and KCl) mimicked the effects of methacholine. Collectively, bronchoconstriction promotes the release of TGF-β, which induces airway smooth muscle remodelling. This study shows that lung slices are a useful *in vitro* model to study mechanisms involved in airway remodelling.

## Introduction

Airway remodelling is an important pathological characteristic of chronic asthma [1]. Airway remodelling encompasses all structural alterations of the airways, including remodelling of the airway smooth muscle layer which is one of the most striking pathological features of chronic asthma [2]. Remodelling of the airway smooth muscle layer has been suggested to be a major cause of airflow obstruction in asthma [3], [4]. The mechanisms underlying this pathology are, however, still unclear.

During bronchoconstriction, airways are subjected to mechanical forces, which promote gene expression and growth factor release in resident cells [5]. As such, mechanical forces could promote tissue remodelling. For example, compression of airway epithelial cells leads to features of remodelling, including upregulation of gene expression of TGF-β and protein expression of fibronectin [5]–[8]. Grainge et al. demonstrated that bronchoconstriction induced by repeated methacholine challenges can induce features of airway remodelling in mild asthma patients [9], including an increase in epithelial TGF-β expression and an increase in subepithelial collagen deposition compared to saline-challenged controls. Moreover, we have previously demonstrated that treatment of sensitized guinea pigs with the anticholinergic bronchodilator drug tiotropium inhibits airway remodelling induced by repeated allergen challenge [10], [11]. These findings have provided new insights into the causality of the relationship between persistent airflow obstruction and airway remodelling in asthma.

The multifunctional cytokine TGF-β plays an important role in airway remodelling [12]. In the airways of asthmatics, this pro-fibrotic cytokine is highly expressed, particularly in epithelial cells and in eosinophils [12], [13]. In airway smooth muscle cells, TGF-β_1_ can induce proliferation [12], [14], [15] as well as increased expression of contractile protein markers, such as sm-α-actin and calponin through both transcriptional and translational control [13], [14]. Activation of serine/threonine kinase receptors by TGF-β_1_ will provoke phosphorylation of Smad 2/3 [16], and promotes the nuclear localization of serum response factor, which cooperatively regulate the transcriptional activity for smooth muscle specific genes [17]–[19]. Furthermore, TGF-β_1_ can regulate cell proliferation of airway epithelial cells and fibroblasts, but also cell differentiation and the synthesis of extracellular matrix proteins in these cells [20]. Although evidence exist that TGF-β is upregulated after mechanical compression of airway epithelial cells and is involved in tissue remodelling, a direct link between bronchoconstriction and TGF-β-induced airway remodelling has not been demonstrated yet.

Precision-cut lung slices have been proven a valuable *in vitro* tool in pharmacological research and drug development [21]. Lung slices have various advantages compared to airway smooth muscle cell cultures, as all lung cell types are present and cell-cell contacts and cell-matrix interactions are preserved, which are important regulators in bronchoconstriction and airway remodelling [22]. Additionally, the profound loss of contractile capacity of airway smooth muscle cells in culture, associated with a loss of sm-myosin expression and contractile receptors, is avoided [23], [24]. Moreover, a large number of lung slices can be prepared from the lungs of a single animal, which allows the direct comparison of experimental treatments with a control from the same animal [24]. The use of precision-cut lung slices from guinea pigs has additional advantages. For example, Ressmeyer et al., showed that airway responsiveness to methacholine was almost identical for precision-cut lung slices from humans and guinea pigs [24]. Moreover, in contrast to other small experimental animals, there are great anatomical and functional similarities between guinea pig and human airways including the presence of small airways [4].

Therefore, in the present study, precision-cut lung slices were used as an *in vitro* model to study mechanisms of bronchoconstriction-induced airway remodelling. Precision-cut lung slices from guinea pigs were stimulated with contractile agonists such as methacholine, histamine and potassium chloride, but also with the pleiotropic cytokine TGF-β_1_. Contractile protein expression was studied in response to these stimuli as a marker of airway remodelling. Using specific inhibitors, the mechanisms inducing an increase in contractile protein expression by bronchoconstriction were studied and were found to be dependent on mechanically-induced release of endogenous TGF-β.

## Materials and Methods

### Animals

Outbred, male, specified pathogen-free Dunkin Hartley guinea pigs (Harlan, Heathfield, UK) (740±85 g) were used. All protocols described in this study were approved by the University of Groningen Committee for Animal Experimentation, Groningen, The Netherlands. The animals were housed under a 12 hour light/dark cycle in a temperature- and humidity-controlled room with food and tap water ad libitum.

### Precision-cut lung slices

Precision-cut lung slices were prepared as decribed in [24]. In short, after euthanization by injection with pentobarbital (Euthasol 20%, Produlab Pharma, Raamsdonksveer, the Netherlands) the trachea was cannulated, and the animal was ex-sanguinated via the aorta abdominalis. Lungs were filled through the cannula with a low melting-point agarose solution (1,5% final concentration (Gerbu Biotechnik GmbH, Wieblingen, Germany) in CaCl_2_ (0.9 mM), MgSO_4_ (0.4 mM), KCl (2.7 mM), NaCl (58.2 mM), NaH_2_PO_4_ (0.6 mM), glucose (8.4 mM), NaHCO_3_ (13 mM), Hepes (12.6 mM), sodium pyruvate (0.5 mM), glutamine (1 mM), MEM-amino acids mixture (1∶50), and MEM-vitamins mixture (1∶100), pH = 7.2). Subsequently, lungs were placed on ice for at least 30 min, in order to solidify the agarose for slicing. Tissue cores were prepared from the lobes using a rotating sharpened metal tube (diameter 15 mm), followed by slicing the tissue in medium composed of CaCl_2_ (1.8 mM), MgSO_4_ (0.8 mM), KCl (5.4 mM), NaCl (116.4 mM), NaH_2_PO_4_ (1.2 mM), glucose (16.7 mM), NaHCO_3_ (26.1 mM), Hepes (25.2 mM), pH = 7.2, using a tissue slicer (Compresstome™ VF-300 microtome, Precisionary Instruments, San Jose CA, USA). Lung slices were cut at a thickness of 500 µm.

### Culture medium

Before stimulation, lung slices were incubated individually in 24 well-plates in minimal essential medium composed of CaCl_2_ (1.8 mM), MgSO_4_ (0.8 mM), KCl (5.4 mM), NaCl (116.4 mM), NaH_2_PO_4_ (1.2 mM), glucose (16.7 mM), NaHCO_3_ (26.1 mM), Hepes (25.2 mM), sodium pyruvate (1 mM), glutamine (2 mM), MEM-amino acids mixture (1∶50), and MEM-vitamins mixture (1∶100), pH = 7.2, at 37°C in a humid atmosphere. In order to remove the agarose and cell debris from the tissue, medium was refreshed after 30 min, followed by 2 washes every hour. During the experiments, the lung slices were incubated in Dulbecco's Modification of Eagle's Medium (DMEM) supplemented with sodium pyruvate (1 mM), non-essential amino acid mixture (1∶100), gentamycin (45 µg/mL), penicillin (100 U/mL), streptomycin (100 µg/mL) and amphotericin B (1.5 µg/mL) at 37°C–5% CO_2_.

### Lactate dehydrogenase release

To assess the viability of the lung slices, the amount of lactate dehydrogenase (LDH) released from the slices into the incubation medium relative to the total slice content was measured. Slices were incubated in 6 well-plates (2 slices/well) in 4 mL incubation medium for 1, 2, 3 or 4 days. Maximal LDH release was determined by lysing 2 slices with 1% Triton X-100 for 30 min at 37°C at the beginning of the experiment. Supernatants were stored −80°C. LDH release in the supernatant was determined by routine clinical chemistry at the UMCG (Groningen, The Netherlands) using the Roche/Hitachi Modular system (Roche, Mannheim, Germany).

### Mitochondrial activity assay

Mitochondrial activity, as an additional measure of tissue viability, was assessed by conversion of Alamar blue into its reduced form, as described previously [25]. Lung slices were incubated with Hanks' balanced salt solution, containing 10% Alamar blue solution (BioSource, Camarillo, CA), for at least 30 min at 37°C-5% CO_2_ [25].

### Treatment of lung slices

Lung slices were cultured in 6-well plates, using 2 slices per well. The slices were stimulated with methacholine (10 µM), TGF-β_1_ (0.1, 0.2, 1 and 2 ng/mL), KCl (60 mM) or histamine (1 µM) for 1 or 2 days continuously, as indicated. Where mentioned, lung slices were pre-incubated with the inhibitor of actin polymerization latrunculin A (0.3 µM) or the selective inhibitor of the TGF-β type I receptor activin receptor-like kinase ALK5, SB431542 (0.3 µM) for 30 min.

### Stimulation of MRC-5 fibroblasts by conditioned media of stimulated lung slices

MRC-5 lung fibroblasts were cultured in Ham's F12 medium supplemented with 10% foetal bovine serum (FBS), L-glutamine (2 mM), streptomycin (100 µg/L) and penicillin (100 U/mL). For each experiment, cells were grown to confluence and subsequently culture medium was substituted with Ham's F12 medium supplemented with 0.5% FBS, L-glutamine and antibiotics for a period of 24 hours. Thereafter, cells were stimulated for 1 h with TGF-β_1_ (2 ng/mL), methacholine (10 µM) or with conditioned media obtained from incubated lung slices. Conditioned media used were taken from control slices and from slices treated with TGF-β_1_ (2 ng/mL) or MCh (10 µM), both in the presence and absence of the inhibitors latrunculin A (0.3 µM) or SB431542 (0.3 µM), for 48 h.

### Immunofluorescence

Lung slices were stimulated in 6 well-plates, using 2 slices per well. Slices were stimulated with methacholine (10 µM) or TGF-β_1_ (2 ng/mL) for 2 days continuously. After washing twice with cytoskeleton buffer (CB: MES (10 mM), NaCl (150 mM), EGTA (5 mM), MgCl_2_ (5 mM), and glucose (5 mM) at pH = 6.1), the lung slices were fixed with CB containing 3% paraformaldehyde (PFA) for 15 min. The slices were subsequently incubated with CB buffer containing 3% PFA and 0.3% Triton-X-100 for 5 min, followed by an additional 2 washes with CB buffer. Lung slices were then blocked for 1 h in cyto-TBS (Tris-base (20 mM), NaCl (154 mM), EGTA (2.0 mM) and MgCl_2_ (2.0 mM), pH = 7.2), supplemented with BSA (1%) and normal donkey serum (2%). After that, lung slices were stained with mouse sm-myosin antibody overnight at 4°C. After washing 3 times with cyto-TBS containing 0.1% Tween-20 (cyto-TBS-T) for 10 min, incubation with the secondary antibody (Cy3-mouse, 1∶50 in cyto-TBS-T) was performed during 3 h at room temperature. Lung slices were then washed 4 times for 15 min in cyto-TBS-T, followed by 4 washes with ultra-pure water. The slides were mounted with ProLong Gold anti-fade reagent (Invitrogen, Breda, The Netherlands). After staining, the slides were analysed using a Leica TCSSP2 confocal microscope. All conditions within one experiment were analysed in the same session using identical microscopic settings. Excitation wavelength was 543 nm.

### Western Blotting

Lung slices or MRC-5 cells were washed once with ice-cold phosphate-buffered saline (PBS, composition: NaCl (140 mM), KCl (2.6 mM), KH_2_PO_4_ (1.4 mM), Na_2_HPO_4_ (8.1 mM), pH 7.4), followed by lysis using ice-cold SDS-lysis buffer (Tris-HCl (62.5 mM), SDS (2%), NaF (1 mM), Na_3_VO_4_ (1 mM), aprotinin (10 µg/mL), leupeptin (10 µg/mL), pepstatin A (7 µg/mL) at pH 8.0). Equal amounts of protein, determined by Pierce protein determination according to the manufacturer's instructions, were separated on SDS polyacrylamide gels and transferred onto nitrocellulose, followed by standard immunoblotting techniques. All bands were normalized either to β-actin or to total Smad 2/3 expression.

### Antibodies and reagents

Methacholine chloride was purchased from ICN Biomedicals (Zoetermeer, the Netherlands). Human recombinant TGF-β_1_ was obtained from R&D systems (Abingdon, UK). Mouse anti-α smooth muscle actin (sm-α-actin) antibody, mouse anti-calponin antibody, mouse anti-β-actin (β-actin) antibody, horseradish peroxidase (HRP)-conjugated rabbit anti-mouse IgG antibody, histamine were purchased from Sigma-Aldrich (Zwijndrecht, The Netherlands). Mouse anti-smooth muscle myosin (sm-myosin) was purchased from Neomarkers (Immunologics, Duiven, The Netherlands). Cy3-conjugated secondary antibody was obtained from Jackson ImmunoResearch (West Grove PA, USA). Rabbit anti-phospho-Smad3 (Ser423/425) was from Cell Signaling Technology (Beverly, MA, USA). Rabbit anti-total Smad 2/3 (FL-425) was purchased at Santa Cruz Biotechnology (Heidelberg, Germany). The inhibitors latrunculin A and SB431542 were purchased from Tocris Biosciences (Bristol, UK). All other chemicals were of analytical grade.

### Data analysis

Data are presented as mean values ± SEM. Statistical significance was determined by paired Student's *t*-test with two-tailed distribution or one-way ANOVA for paired observations, followed by a Newman-Keuls multiple comparisons test when appropriate. Data were considered statistically significant if p<0.05.

## Results

### Lung slice viability

**Figure 1:**
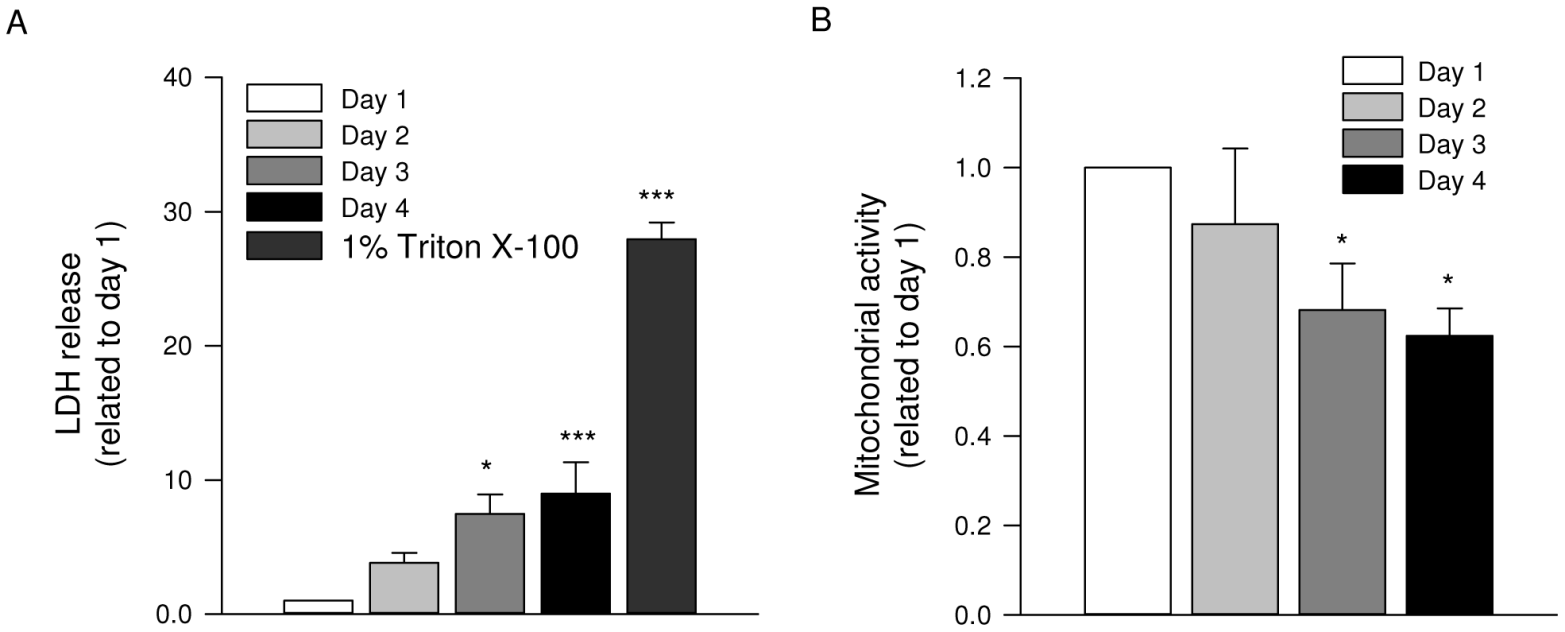
Lung slice viability. LDH release from lung slices after 1, 2, 3 and 4 days of culture. Maximal LDH content of the slices was established by lysis with 1% Triton X-100 (A). Data shown are the means ± SE of 3–7 independent experiments. Mitochondrial activity in lung slices after 1, 2, 3 and 4 days of culture (B). Data shown are the means ± SE of 5 independent experiments. ^*^: p<0.05 and ^***^: p<0.001 compared to basal (one-way ANOVA, posthoc Newman-Keuls).

To determine the viability of cultured precision-cut lung slices, LDH release and mitochondrial activity were assessed after 1, 2, 3 and 4 days of incubation. We observed a significant increase in LDH release over time; however, LDH release was still below 15% of maximal content on day 2 (Figure 1A) as observed previously in Ressmeyer et al. [24]. The time course of LDH release was closely paralleled by that of the mitochondrial activity assay, which demonstrated a significant reduction in activity starting on day 3 (Figure 1B). Based on these data, we used lung slices that were cultured for up to 2 days.

### Contractile protein expression in response to TGF-β_1_

**Figure 2:**
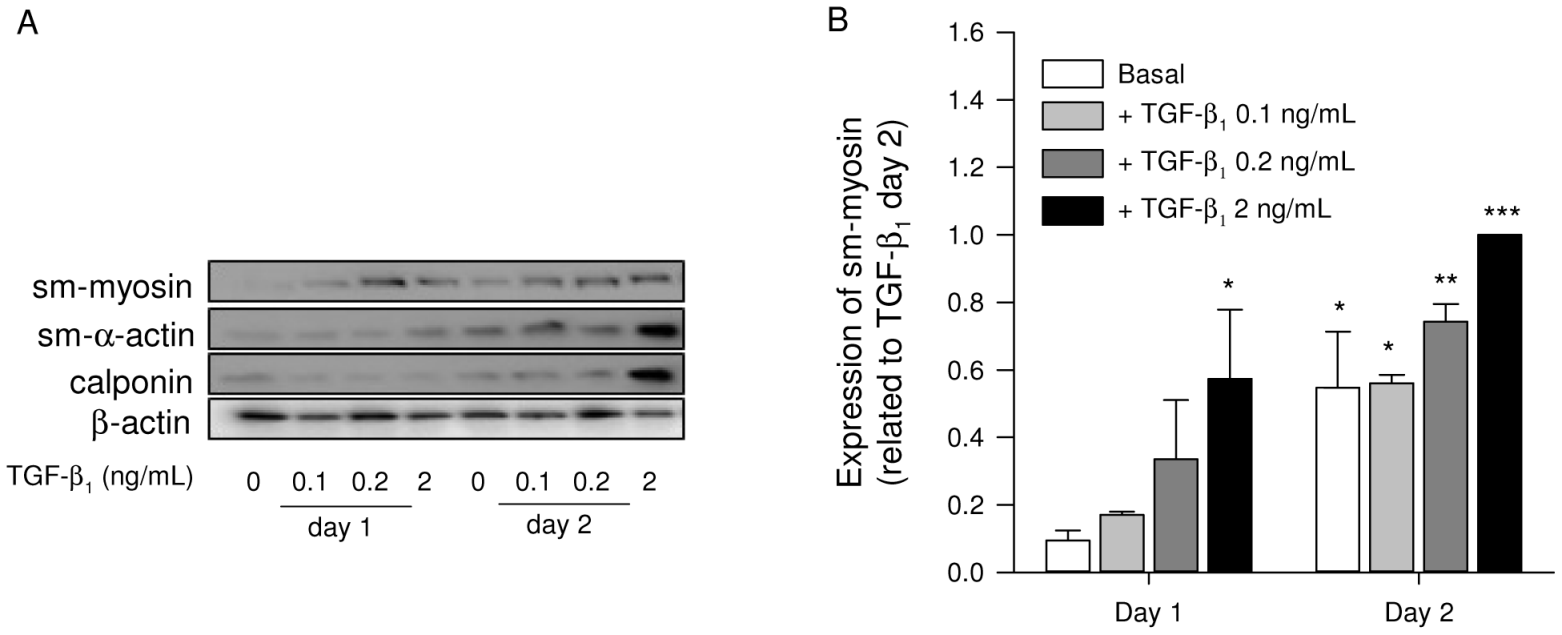
Concentration and time-dependent effects of TGF-β_1_ on contractile protein expression. Lung slices were treated in a time- and concentration-manner with TGF-β_1_ (0, 0.1, 0.2, 2 ng/mL) for 1 or 2 days, as indicated. Lung slice lysates were analysed for sm-myosin, sm-α-actin, or calponin, using β-actin as a loading control. Representative blots of TGF-β_1_-induced contractile protein expression (A). Densitometric analysis of sm-myosin expression (B). Data shown are means ± SE of 3 independent experiments. ^*^: p<0.05, ^**^: p<0.01, and ^***^: p<0.001 compared to basal (one-way ANOVA, posthoc Newman-Keuls).

TGF-β_1_ is a potent cytokine that induces cellular biological processes leading to airway remodelling [12]. Therefore, in order to investigate the usefulness of lung slices as an *in vitro* model to study airway remodelling, we studied the effect of TGF-β_1_ treatment on contractile protein expression in lung slices. Lung slices were incubated in a dose- and time-dependent manner for 1 or 2 days in the presence or absence of TGF-β_1_ (0.1, 0.2, 2 ng/mL) and analysed for the expression of sm-myosin, sm-α-actin and calponin. We observed that TGF-β_1_ induced a time- and a concentration-dependent increase in the expression of sm-myosin, sm-α-actin and calponin (Figure 2A–B). The maximal response measured for sm-myosin was obtained by incubating the slices with 2 ng/mL TGF-β_1_ for 2 days continuously (Figure 2B). This condition was chosen for further experiments with this cytokine.

### Contractile protein expression in response to methacholine-induced bronchoconstriction

**Figure 3:**
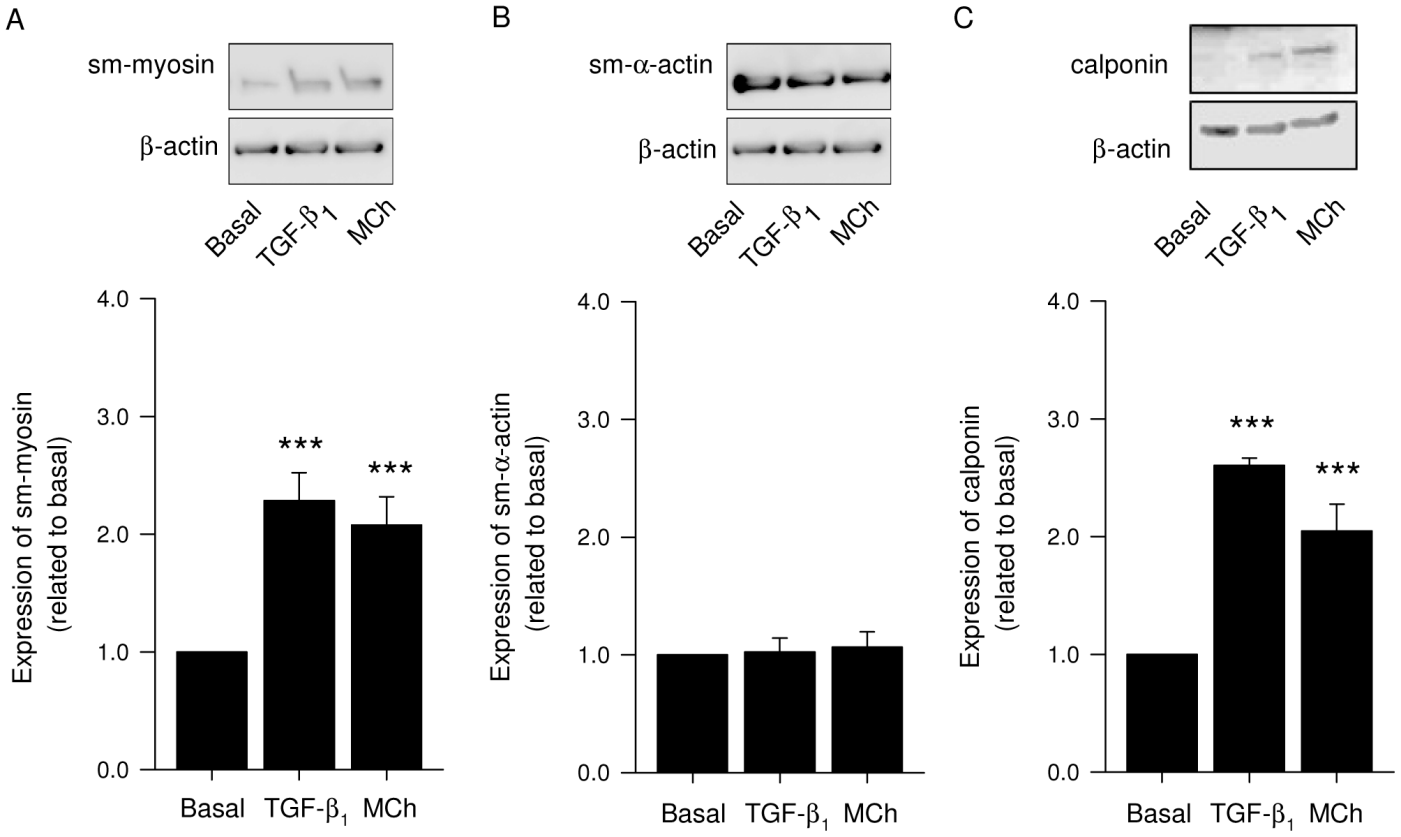
Methacholine stimulation induces contractile protein expression in lung slices. Lung slices were treated with TGF-β_1_ (2 ng/mL), methacholine (MCh; 10 µM) or medium (basal) for 2 days. Lung slice lysates were then analysed for the presence of sm-myosin (B), sm-α-actin (C), or calponin (D) using β-actin as a loading control. Representative blots are shown in (A). Data shown in graphs are the means ± SE of 3–4 independent experiments. ^***^: p<0.001 compared to basal (one-way ANOVA, posthoc Newman-Keuls).

We next determined whether bronchoconstriction also induced the expression of contractile proteins. Stimulation of lung slices with 10 µM methacholine for 2 days resulted in an increased expression of sm-myosin and calponin (1,69±0.23- and 2.89±0.59- fold induction, respectively). However, methacholine did not induce an increase in the expression of sm-α-actin (Figure 3A–D).

**Figure 4:**
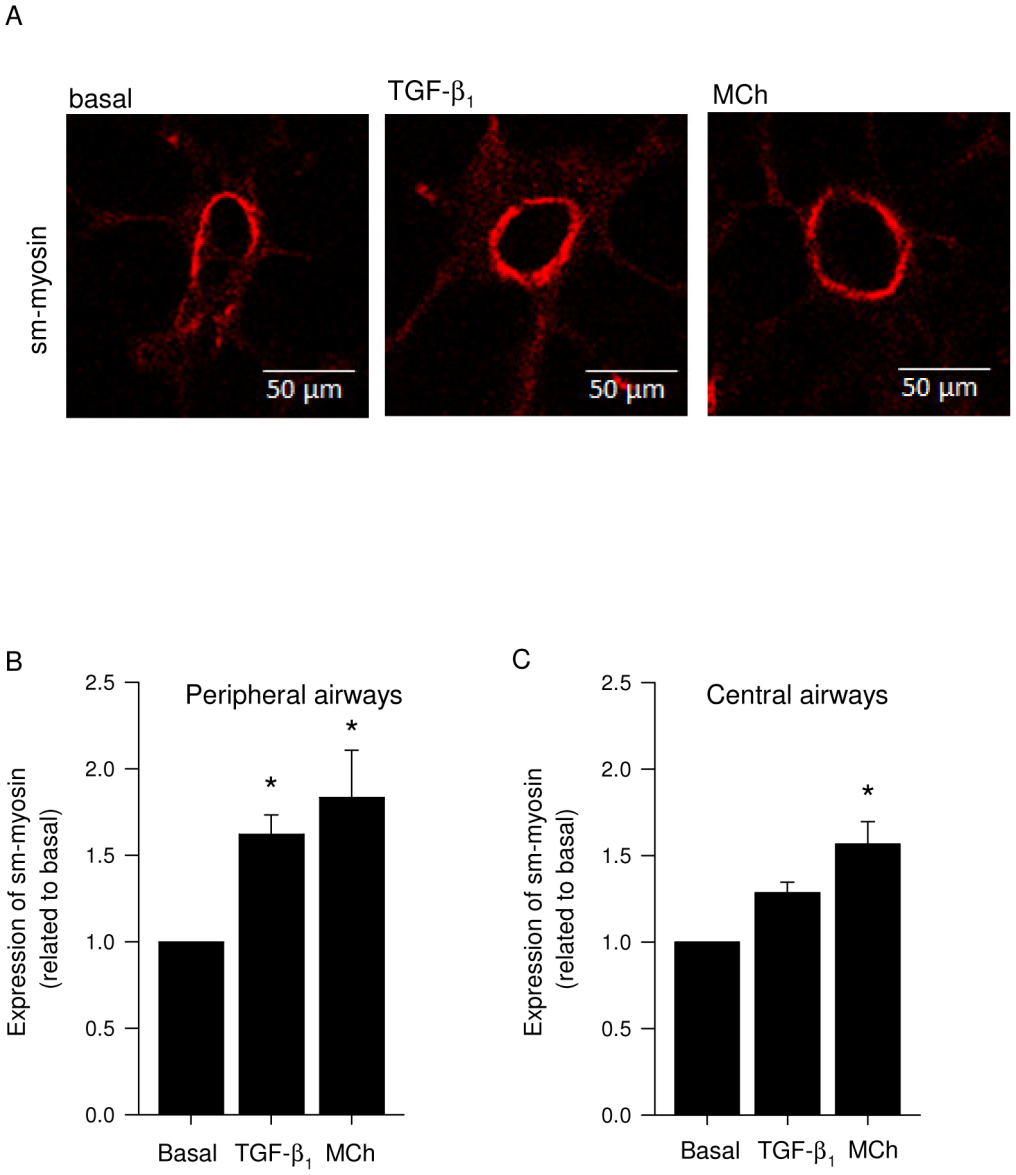
Localization of sm-myosin expression after TGF-β_1_ and methacholine treatment. Lung slices were treated with TGF-β_1_ (2 ng/mL), methacholine (MCh; 10 µM) or medium (basal) for 2 days. Lung slices were then fixed, stained for sm-myosin and analysed by confocal immunofluorescence microscopy. The images shown in (A) are taken from the peripheral airways (diameter smaller than 100 µm). Staining intensity within the muscle bundle was quantified and data shown from the peripheral airways (B) and central airways (diameter larger than 400 µm; C) are the means ± SE of 3 independent experiments. ^*^: p<0.05 compared to basal (one-way ANOVA, posthoc Newman-Keuls).

In view of these data, we further investigated the localization of increased contractile protein expression induced by TGF-β_1_ and methacholine in the airways. Therefore, lung slices incubated with either TGF-β_1_ (2 ng/mL) or methacholine (10 µM) were stained immuno-cytochemically for sm-myosin and visualized using confocal fluorescence microscopy. Staining intensity within the smooth muscle bundle was quantified (Figure 4A–C). To distinguish peripheral airways from central airways, the diameter of the airways was measured. Airways with a diameter under 100 µm were considered as peripheral airways and with a diameter above 400 µm as central airways. Increased expression of sm-myosin, but not of sm-α-actin (data not shown) in response to methacholine and TGF-β_1_ as visualized by confocal fluorescence microscopy was found in peripheral airways with a diameter smaller than 100 µM (1.8±0.3 and 1.6±0.1 fold-induction for methacholine and TGF-β_1_, respectively, Figure 4A–B), and in the central airways, with a diameter larger than 400 µM (1.6±0.1 fold-induction for methacholine Figure 4C).

### Mechanisms of bronchoconstriction-induced airway remodelling

**Figure 5:**
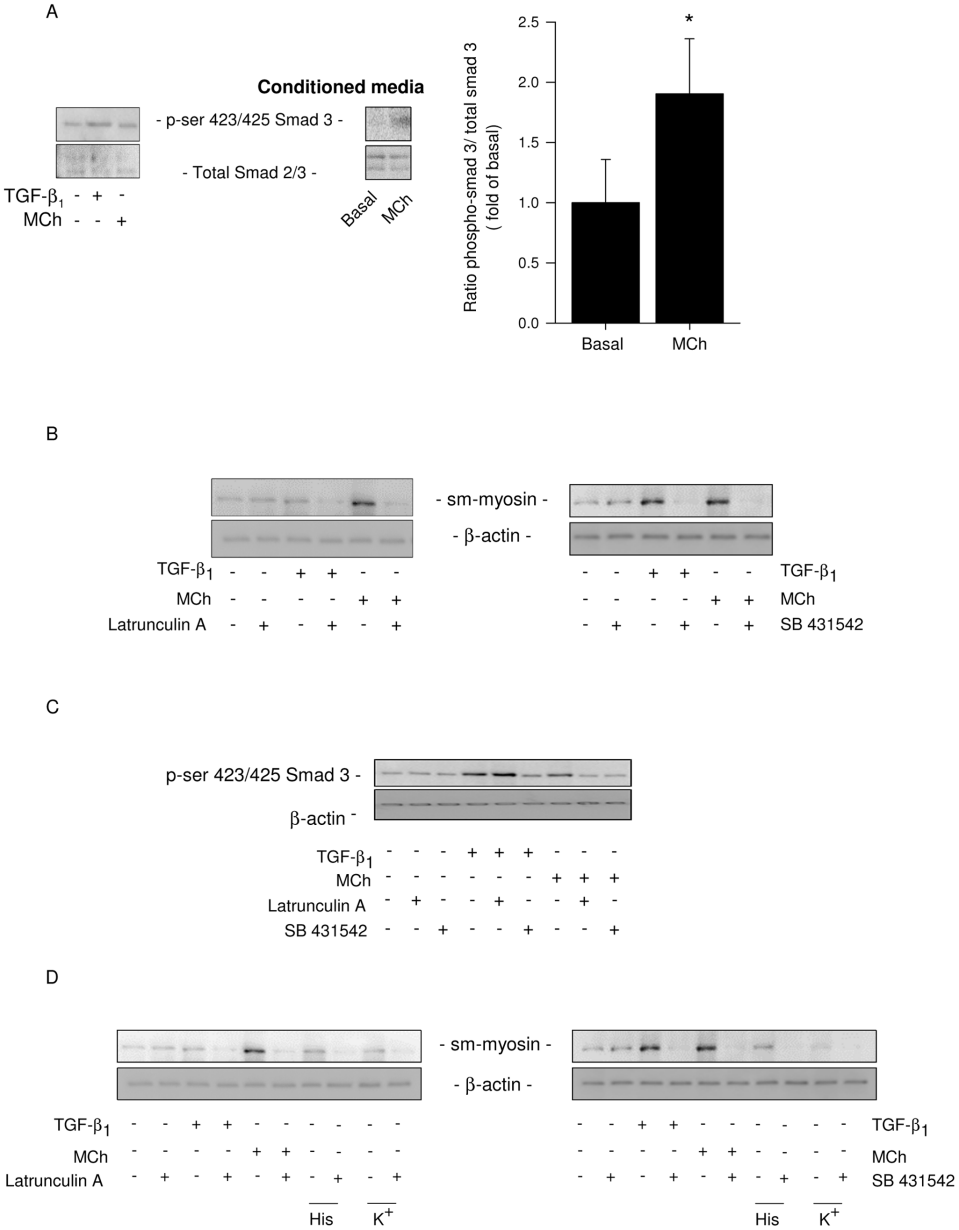
Bronchoconstriction induces the release of biologically active TGF-β leading to contractile protein expression. Human MRC-5 fibroblasts were stimulated for 1 hour with TGF-β_1_ (2 ng/mL), methacholine (MCh; 10 µM) or medium (basal), or with conditioned media obtained from lung slice cultures treated for 2 days with and without 10 µM methacholine. MRC-5 cell lysates were analysed for phosphorylated (ser 423/425) and total Smad-3. Representative blots and quantified data of Smad-3 phosphorylation in response to conditioned media are shown in (A). Data shown are the means ± SE of 4 independent experiments. ^*^: p<0.05, compared to basal (paired Student's *t*-test with two-tailed distribution). Lung slices were pre-treated with latrunculin A (0.3 µM), SB431542 (0.3 µM), or medium (basal) for 30 min, followed by 2 days of treatment with methacholine (MCh; 10 µM), TGF-β_1_ (2 ng/mL), or medium (basal) (B). Lung slice lysates were analysed for the presence of sm-myosin, using ß-actin as a loading control. Blots shown are representative of 3 experiments. Human MRC-5 fibroblasts were stimulated for 1 hour with conditioned media obtained from lung slice cultures after treatment with methacholine (MCh; 10 µM), TGF-β (2 ng/mL) or medium (basal), in the absence and presence of latrunculin A (0.3 µM) or SB431542 (0.3 µM) (C). MRC-5 cell lysates were analysed for phosphorylated (ser 423/425) and total Smad-3. Blots shown are representative of 3 experiments. Lung slices were pre-incubated with latrunculin A (0.3 µM), SB431542 (0.3 µM), or medium (basal) for 30 min, followed by 2 days stimulation with methacholine (MCh; 10 µM), TGF-β_1_ (2 ng/mL), histamine (His, 1 µM), KCl (K^+^, 60 mM) or medium (basal) (D). Lung slice lysates were analysed for sm-myosin, using β-actin as a loading control. Blots shown are representative of 3 experiments.

Bronchoconstriction induced by methacholine causes an increase in the release of epithelial TGF-β in asthma patients [11]. Also, co-culture of airway smooth muscle cells and epithelial cells causes the release of biologically active TGF-β in response to contractile agonists such as lysophosphatidic acid and methacholine [26]. Therefore, the increase in contractile protein expression we observed in response to bronchoconstriction may be due to the release of TGF-β by lung slices. To establish whether biologically active TGF-β is involved in the increased contractile protein expression in response to methacholine, biologically active TGF-β was determined in conditioned media from stimulated precision-cut lung slices, using Smad-3 phosphorylation in human MRC-5 fibroblasts as a bio-assay. Human MRC-5 fibroblasts were incubated for 1 hour with the conditioned media obtained from methacholine-stimulated lung slices, followed by analysis of phosphorylated Smad-3, which is specifically activated by TGF-β. The phosphorylation of Smad-3 was significantly increased by conditioned media from methacholine-stimulated lung slices compared to basal controls (Figure 5A). As controls, the direct phosphorylation of Smad-3 in response to TGF-β_1_ and methacholine in human MRC-5 fibroblasts were investigated. TGF-β_1_, but not methacholine, induced direct phosphorylation of Smad-3 in the MRC-5 cells (Figure 5A), confirming that the effect of methacholine was due to the release of biologically active TGF-β from the lung slices.

To investigate whether bronchoconstriction is involved in methacholine-induced contractile protein expression and release of TGF-β, we inhibited actin polymerization with latrunculin A. Latrunculin A prevented the increase in sm-myosin expression in response to methacholine and TGF-β_1_ (Figure 5B). In addition, the release of bioactive TGF-β by methacholine was inhibited by latrunculin A, whereas latrunculin A had no direct effect on TGF-β_1_-induced Smad-3 phosphorylation (Figure 5C). Moreover, inhibition of TGF-β type I receptor kinase with SB431542 prevented the increase in expression of sm-myosin induced by either TGF-β_1_ or methacholine (Figure 5B). Collectively, these findings indicate that bronchoconstriction induced by methacholine leads to the release of TGF-β, which enhances the expression of contractile proteins.

To establish whether the increase in contractile protein expression was only seen with the contractile agonist methacholine, we stimulated lung slices also with the contractile agonists histamine (His, 1 µM) and potassium chloride (KCl, 60 mM) in the presence or absence of the inhibitors latrunculin A or SB431542. The expression of sm-myosin was enhanced in response to the contractile agonists methacholine, histamine and KCl, and these responses were similarly inhibited by latrunculin A and SB431542 (Figure 5D). This suggests that bronchoconstriction leads to the release of TGF-β inducing the expression of the contractile protein sm-myosin irrespective of the contractile agonist used.

## Discussion

After validation of lung slices as an *in vitro* model for TGF-β induced airway smooth muscle remodelling, we studied the mechanisms involved in the induction of airway remodelling in response to bronchoconstriction. We show that bronchoconstriction induced by contractile agonists including methacholine, histamine and KCl, stimulates the release of TGF-β from lung tissue, which leads to an enhanced expression of contractile phenotype markers by the airway smooth muscle, similar to what is observed in patients with asthma [27], [28].

Airway remodelling is a multicellular process, in which structural cell-cell interactions and cell-matrix interactions play a major regulatory role [29]. Therefore, lung slices appear to be a useful *in vitro* model to study multicellular remodelling processes as most cell-cell contacts, and the cell-matrix interactions are preserved in this model. This has already been established for other organ systems, such as liver slices, in which ethanol or CCl_4_ induced liver fibrosis can be adequately mimicked [30]–[32]. Another advantage of lung slices in culture is that the loss of sm-myosin expression that is typical for cultured airway smooth muscle cells is not observed. The choice for guinea pig lung slices in the present study is based on the observations that airway responsiveness to methacholine in guinea pig lung slices is very similar to that in human tissue [4]. Furthermore, guinea pigs have great anatomical and functional similarities compared to human airways including the presence of small airways in contrast to other animals [24].

The use of lung slices also has some limitations. The major disadvantages of lung slices as an *in vitro* model include the lack of circulation and oedema formation [33], which interferes with the ability to clear soluble factors, including TGF-β. Also, relevant to the present study, tachyphylaxis to sustained bronchoconstriction could occur during 2 days of culture with bronchoconstricting agonists. However, as bronchoconstriction remained visible after 2 days of culture, and as other contractile agonists, including histamine and potassium chloride produced very similar results, we believe that even though tachyphylaxis may have occurred to some extent, its impact on the overall conclusion is small. Furthermore, the period during which lung slices can be maintained in culture is limited. Nonetheless, after 2 days of culture, we still measured low levels of LDH release and no decline in mitochondrial activity, indicating good viability. Very importantly, we observed an increase in contractile protein expression in response to TGF-β_1_ as a marker of airway remodelling, which underscores the usefulness of this culture system. Overall, this model offers a great perspective for future experiments, particularly in studying research questions in which intact cell-cell and cell-matrix interactions are essential.

The pleiotropic cytokine TGF-β is an important growth factor involved in airway remodelling processes in asthma [12]. Increased expression of TGF-β is found in lung tissue and bronchoalveolar lavage fluid of patients with asthma [34]. TGF-β promotes important aspects of airway remodelling, including maturation of airway smooth muscle cells characterized by increased expression of contractile phenotype marker proteins [35]. Indeed, we previously showed that TGF-β induces increased expression of the contractile phenotype markers sm-α-actin and calponin in human airway smooth muscle cells in a time-dependent manner, which was synergistically enhanced by muscarinic receptor stimulation [13], [14]. Patients with chronic asthma show an increase in sm-α-actin and myosin light chain kinase staining in the airways [27], [28]. In agreement with these data, we show that lung slices in culture exhibit a time- and concentration-dependent increase of contractile phenotype markers including sm-myosin, in response to TGF-β. This implies that TGF-β responses in lung slices are similar to those observed in airway smooth muscle cell systems.

Our studies provide important insights into the mechanisms behind the induction of contractile protein expression in response to bronchoconstricting agents. Our previous studies using cultured airway smooth muscle cells showed that muscarinic receptor stimulation had no effect on cell proliferation, cytokine production or contractile protein expression by its own but required functional interactions with growth factors (e.g. PDGF-AB, TGF-β), cytokines (e.g. TNF-α) or cigarette smoke extract to induce these cellular responses [13], [36]–[39]. Cooperative regulation of contractile protein expression by TGF-β and methacholine was found to be associated with enhanced GSK-3 and 4E-BP1 phosphorylation [13]. The present study shows that in lung slices, in which cell-cell interactions and contractility are preserved, bronchoconstriction induced by methacholine is sufficient to promote the expression of contractile proteins, including sm-myosin and calponin, which is explained by the release of biologically active TGF-β which may functionally interact with methacholine to induce contractile expression. Other bronchoconstricting agents, including histamine and KCl were also sufficient to induce these effects. Notably, bronchoconstriction induced by methacholine did not enhance the expression of the contractile protein sm-α-actin. We previously demonstrated that sm-α-actin positive area in guinea pig airways is larger compared to sm-myosin positive area suggesting that actin positive, myosin negative cells exist within the smooth muscle bundle [10]. Moreover, allergen-challenged guinea pigs show much greater induction of sm-myosin expression in comparison to sm-α-actin [10]. Although we cannot directly compare the effect of allergen with methacholine, the similarity with our current data is remarkable and suggests that sm-α-actin is less susceptible to regulation than sm-myosin in guinea pig airways. The TGF-β response by itself was also affected by the actin polymerization inhibitor latrunculin A, suggesting a basal tone of the airways, leading to the release of TGF-β or a requirement of actin polymerization for the induction of smooth muscle specific gene expression, also in response to TGF-β [40]–[42]. Taken together, this suggests that bronchoconstriction induces the release of biologically active TGF-β, leading to contractile protein expression.

In response to bronchoconstriction induced by methacholine or house dust mite, epithelial-TGF-β levels are increased in patients with asthma [9]. In the airways biopsies of human lung, immunostaining revealed that TGF-β was mainly localized to the bronchial epithelial compartment, and to a lesser extent in smooth muscle cells [43]. Mechanical stimulation of bronchial epithelial cells induced the release of TGF-β, playing an important role in subepithelial processes observed in asthma [8]. In response to static transmembrane pressures, rat epithelial cells also promote the gene expression of TGF-β, endothelin-1 and early growth response-1 [44]. Additionally, mechanical stimulation induces an increase in fibronectin production in epithelial cells [5]. This may suggest that the primary sources of TGF-β in the airways are the epithelial cells, which release this growth factor in response to mechanical stimulation during bronchoconstriction. Nonetheless, the airway smooth muscle may also play an important role as Tatler et al. demonstrated that airway smooth muscle cells can activate TGF-β through α_V_β_5_ integrins in response to bronchoconstrictors *in vitro* [26]. Moreover, blocking the α_V_β_5_ integrins caused a reduction in the increased ASM layer *in vivo* with an ovalbumin-challenged mice model [26]. Furthermore, in response to the contractile agonists lysophosphatidic acid and methacholine, airway smooth muscle cells from asthma patients released TGF-β to a greater extent than airway smooth muscle cells from healthy controls [26]. Therefore, epithelial release of TGF-β and subsequent activation of the latent form into the biologically active form by α_V_β_5_ integrins on airway smooth muscle is a plausible mechanism for the effects of bronchoconstriction observed in our study.

Our findings have important implications for the management of chronic asthma. As bronchoconstriction can promote airway remodelling, the beneficial effects of bronchodilator drugs may exceed their acute effects on lung function. *In vitro* and *in vivo* studies are supporting this hypothesis. In a murine model of asthma, levels of TGF-β were reduced in bronchoalveolar lavage fluid (BALF) by the anticholinergic drug tiotropium. Additionally, tiotropium inhibited the thickening of airway smooth muscle and airway fibrosis in this model [45]. Tiotropium treatment also inhibited the increase in contractile protein expression and thickening of the airway smooth muscle layer, in response to allergen-challenge in guinea pigs [10], [11]. In fact, anticholinergics have multiple anti-inflammatory and anti-remodelling properties in animal studies (for review, see Kistemaker, Oenema el al. [46]), however, the underlying mechanism are still not understood. β-Agonists, which are widely prescribed as bronchodilator drugs to asthmatics, can also reduce, though only partially, TGF-β-induced contractile protein expression in human bronchial smooth muscle cells [47] and other remodelling processes [12].

In conclusion, using lung slices as an *in vitro* model, our findings demonstrate that bronchoconstriction can induce the release of TGF-β which promotes contractile protein expression. Therefore, our data suggest that bronchodilators may have beneficial effects on airway remodelling which should be followed up in future studies. Also our data suggest that the use of precision cut lung slices is a suitable model to study airway remodelling processes in response to bronchoconstriction.
